# The exceptional case of a large extra-uterine, so-called parasitic leiomyoma in a post-menopausal woman with Hashimoto’s thyroiditis

**Published:** 2021-01-08

**Authors:** W Decleer, A Debeuckelaere, F Comhaire

**Affiliations:** Fertility Centre, AZ Jan Palfijn Ghent, Watersportlaan 5, 9000 Ghent, Belgium; Fertility Clinic, Weststraat 16/18, 9880 Aalter, Belgium; Master student Medical Science, KU Leuven, 3000 Leuven, Belgium

**Keywords:** Parasitic leiomyoma, Hashimoto’s thyroiditis, extra-uterine, post-menopausal

## Abstract

A large tumour mass was detected in a 65-year-old patient during a routine gynaecological examination. This patient had been treated for over 10 years with levothyroxine for Hashimoto’s thyroiditis and was also given transdermal oestrogen replacement therapy. Before the operation, detailed imaging by CT scan and MRI was performed. A tumour weighing 1.056 grams and measuring 23x12x7 cm was successfully removed through laparotomy. Histopathology revealed the diagnosis of an extra-uterine, so-called parasitic leiomyoma. Post-surgery recovery was uneventful, but Tibolone treatment was indicated due to disturbing menopausal complaints.

## Introduction

Uterine leiomyomas, also called fibroids, are common benign adenomas, which sometimes cause metrorrhagia. The growth of fibroids is hormone- dependant, but it is possible that they can shrink after the menopause. However, they may also gain volume during oestrogen replacement therapy. Whilst the overwhelming majority of fibroids are situated in the uterus, extra-uterine, parasitic leiomyomas do occasionally occur (Mahmoud et al., 2015). Several publications report a possible association between leiomyomas and thyroid diseases, namely hypothyroidism (Ott et al., 2014), thyroid adenomas (Kim et al., 2010) and fibroadenomas of the breast (Spinos et al., 2007). An association between leiomyomas and autoimmune Hashimoto’s thyroiditis has not been described before. Rare cases of leiomyoma of the thyroid gland (Biankin and Cachia, 1999) or adrenal gland (Goldman and Brodey, 1994, Huei et al., 2017) have been reported.

To the best of our knowledge, no cases have been published combining a large extra-uterine leiomyoma with Hashimoto’s thyroiditis in a post-menopausal patient receiving adequate hormone replacement therapy with thyroid hormone (Levothyroxine) and oestrogen (Oestrogel).

## Case description

### 

The patient was a 65-year-old obese woman with a BMI of 34.8 kg/m2. She was known to suffer from Hashimoto’s thyroiditis, with elevated anti- thyroglobulin antibodies (232 IU/mL), which has been adequately treated for over 10 years. Treatment with 100 μg of levothyroxine resulted in normal hormone concentrations in the blood: TSH: 0.5.mud/L, free T4: 20.2pmol/L, free T3: 4.9 pmol/L.There were no other biological markers of autoimmune disease. Due to insulin resistance combined with hyperinsulinemia, the patient was successfully treated with the combination of Momordica charantia extract and alpha-lipoic acid (Comhaire, 2015), with a normal postprandial C-peptide concentration of 1.67 nmol/L. She had no cardiac, pulmonary, or systemic diseases.

After the birth of her third daughter, she was sterilized by tubal ligature. Due to disturbing menopausal complaints, the patient applied transdermal oestradiol (0.75 mg/day of Oestrogel, Besins, Brussels, Belgium).

In July 2019, the patient was examined by her usual gynaecologist who detected a large intra- abdominal mass, detached from the uterus.

**Figure 1:**
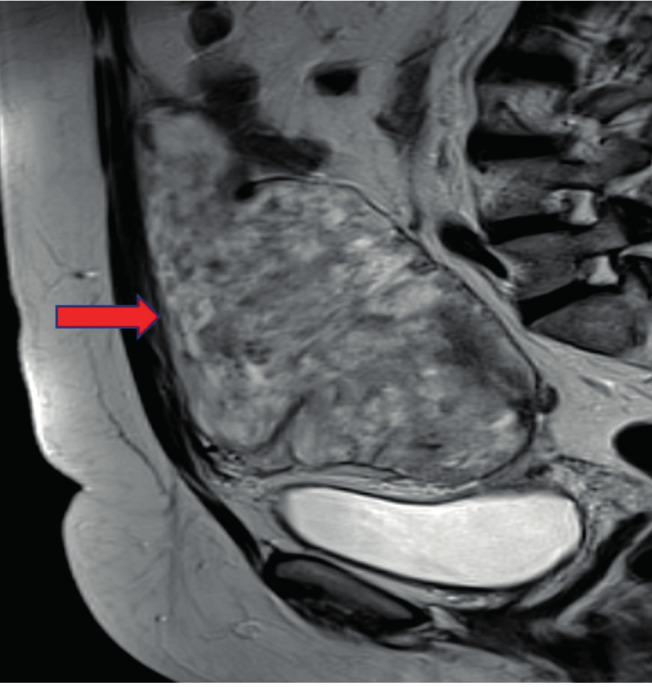
Longitudinal MRI image of the pelvis showing the extensiveness of the parasitic leiomyoma (red arrow: section line between abdominal wall and tumour).

**Figure 2:**
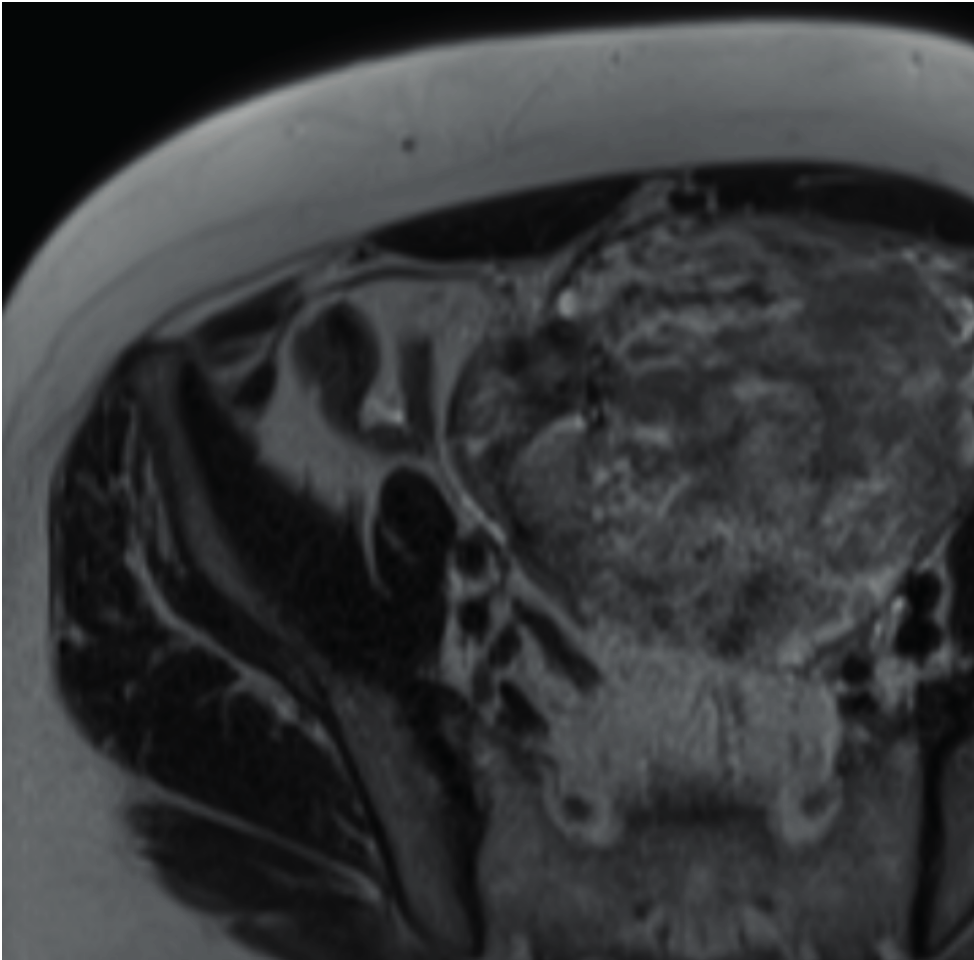
Transversal MRI image of the pelvis showing the extensiveness of the parasitic leiomyoma.

A computerised tomography (CT) scan of the abdomen was performed followed by a magnetic resonance imaging (MRI) scan of the pelvis (Fig.1 & 2) showing the following results:

### Treatment by Surgery

The abdomen was opened by a typical laparotomic incision. The intraperitoneal tumour was attached to the anterior side of the abdominal wall, mostly on the left side. There was no connection with the annexes or the uterus. The uterus itself was moderately enlarged due to a few sub-serosal uterine myomas (<2cm). The peritoneum was opened and the tumour was isolated systematically. The surgical team were able to dissect the tumour from the abdominal wall. Intra-operative frozen section and a gross evaluation of the tumour were performed which revealed a benign stromal tumour of approximately 23 x 12 x 7cm. The left rectus abdominis muscle was slightly damaged during the process of tumour removal, which was immediately restored with Vicryl 2 in separate U-sutures. The peritoneum fascia, subcutis and cutis were closed, and intra-abdominal tubular drainage was placed with the installation of a subfascial Penrose drain.

### Post-surgery

The post-operative course was uneventful, and no complications occurred. Because of this favourable course, the patient was able to leave the hospital after four days. Four weeks later, she was seen at an outpatient consultation (FC) in good health with slight local thickening of the abdominal wall at the left side. No residual tumour residue was detected. The patient complained of disturbing vasomotor hot flushes, cognitive and emotional instability, and sleep disturbance. It was decided to temporarily restart transdermal oestrogen supplementation, which was immediately replaced by long-term Tibolone intake (Gregoriou et al., 1997,Gregoriou et al., 2001).

### Histopathology

Macroscopically, the mass was described as a large nodular tumour of 1056 grams and 23 x 12 x 7 cm. On cut section, it appeared as a solid homogenous light-brown, tan mass. Paraffin sections showed a moderately to poorly cellular tumour, composed of spindle cells, predominantly arranged in bundles. The nuclei were spindle-shaped with blunt ends. Mitoses were few, and no abnormal mitotic figures were seen. The cells had an eosinophilic cytoplasm and were separated by a wide and oedematous interstitium. The sections showed dispersed mast cells and plasmocytes. At the periphery, a pseudocapsule of connective tissue was noticed. Arterial and venous vessels in the capsule were found at the site of the adhesion. Immunohistochemical investigation was performed and resulted in: (1) Smooth muscle actine: positive; (2) Desmin: positive; (3) S100 protein: negative; (4) Ki-67 proliferation marker: <5% positive cell nuclei.

In conclusion, the mass was described as a giant leiomyoma (max. diameter 23 cm) with benign degenerative changes and no signs of malignancy.

## Discussion

Whereas uterine leiomyomas are common gynaecological tumours, extra-uterine so-called parasitic leiomyomas are a rare variant of a pedunculated (uterine) sub-serosal leiomyoma occurring outside the uterus and completely separated from it (Shaukat et al., 2019; Sarmalkar et al., 2016; Grover and Bhalla, 2015). Extra-uterine leiomyoma was first described by Kelly and Cullen in 1909. As per FIGO classification system, so- called parasitic fibroids have been categorized as type 8 leiomyomas with no myometrial involvement and uterine attachment (Berek, 2013).

This mass usually grows intra-peritoneal, more specifically in the pelvis. However, some parasitic myomata have been described in the upper abdomen, on the omentum, in the vagina, sub- or supra-fascial near the port site, sigmoid colon, cervical, retroperitoneal (Cucinella et al., 2011; Epstein et al., 2009; Nezhat and Kho, 2010; Lete et al., 2016),in the thyroid gland (Biankin and Cachia, 1999) and in the adrenal gland (Goldman and Brodey, 1994; Huei et al., 2017).

Parasitic leiomyomas can be either asymptomatic (Salih et al., 2017) or cause complaints such as chronic abdominal pain, urinary dysfunction, abdominal feeling of pressure, palpable mass and rarely acute abdominal pain caused by necrosis or torsion (Cucinella et al., 2011). The diagnosis is often incidentally made upon radiologic examination or at laparoscopic surgery for another reason (Grover and Bhalla, 2015). The diagnosis can pose clinical and diagnostic challenges because these leiomyomas can mimic malignancy due to their unusual location, growth pattern and volume.

The most accurate diagnostic method is a pelvic MRI allowing a detailed description of the tumour and the anatomical relationship with surrounding structures. In addition to these features, MRI may also detect alternative and/or coexistent pelvic or abdominal pathology.

The classic appearance of a typical fibroid on MRI is a well-circumscribed mass with homogeneous T2 hypo-intensity and T1 iso-intensity relative to the myometrium (Bolan and Caserta, 2016). However, only histopathological analysis can confirm the diagnosis of a benign parasitic leiomyoma.

An interesting and rather unique feature of the present case is that the leiomyoma occurred in a patient with Hashimoto’s thyroiditis, but without hypothy roidism. There were no other characteristics of autoimmune disease, but the patient presented with insulin resistance and hyperinsu linemia related to obesity. Insulin is a growth factor and hyperinsulinemiais associated with tumour development (Tsujimoto et al., 2017). Thus, hyperinsulinemia may have contributed to the rapid expansion of this large, though benign tumour mass.

The accepted hypothesis of a parasitic leiomyoma is that they are subserous or stemmed myomata. They may detach from the uterus as a result of traction and torsion and be implanted elsewhere (Kho and Nezhat, 2009; Garrido et al., 2018; Jayashree and Deepthi, 2016). This is classified as a primary myoma (Sarmalkar et al., 2016). A parasitic abdominal leiomyoma can be caused spontaneously, but it can also be iatrogenic. For example, because of seeding during a myoma morcellation, which is the case in 44% of the patients and is called a secondary parasitic myoma (Lete et al., 2016; Sarmalkar et al., 2016; Kho and Nezhat, 2009; Garrido et al., 2018). The incidence of these secondary parasitic leiomyomata seems to be 0.12-1.2% (Cucinella et al., 2011; Leren et al., 2012; Van der Meulen et al., 2016). After torsion or morcellation, a part of the myoma can be seeded in the abdominal wall or the peritoneum and by developing an auxiliary (often omental or mesenteric) blood supply (revascularization), this leiomyoma can grow in an extra-uterine location (Grover and Ballha, 2015; Verberg et al., 2013).

## Differential diagnosis

Table I displays the differential diagnosis of a parasitic leiomyoma.

**Table I:** The differential diagnosis of a parasitic leiomyoma.

Name	Tissue type / characteristics	Typical location
Leiomyomatosis peritonealisdis seminata	Smooth muscle	Subcoelo matous mesenchyma
Intravenous leiomyomatosis	Smooth muscle	Intraluminal in intrauterine or systemic veins
Benign metastatic leiomyoma	Extra-uterine leiomyoma-like nodules	Pulmonary
Retroperitoneal leiomyosarcoma	Central necrosis, invasive growth and a more heterogeneous appearance	Retroperitoneal
Peritoneal carcinomatosis	Presence of ascites and weight loss	Peritoneal
Primary peritoneal mesothelioma	In men, the bladder, bowel, pancreas and/or liver	Peritoneal
Lymphoma	Lymphadenopathy and non-calcified lymph nodes	Retroperitoneal
Fibrotic type of peritoneal tuberculosis	Necrotic mesenteric lymphadenopathy	Peritoneal

In this case, the diagnosis leiomyomatosis peritonealis disseminata could be ruled out because its origin is smooth muscle metaplasia in the subcoelomatous mesenchyma, which was not the case in this patient. Furthermore, there were no diffuse vascular peritoneal nodules visualized on imaging (Leteet al., 2016; Fasih et al., 2008).

Intravenous leiomyomatosis was discounted as it originates, just like leiomyomatosis peritonealis disseminata, from smooth muscle tissue and should present itself intraluminal in intrauterine or systemic veins (often in the vena cava) (Leteet al., 2016; Fasih et al., 2008).

A benign metastatic leiomyoma is normally characterized by solitary extra uterine leiomyoma- like nodules, especially pulmonary, and are often found in women with a pelvic surgical history (Leteet al., 2016; Fasih et al., 2008). This diagnosis was also less likely because these conditions were not met.

Retroperitoneal leiomyosarcoma is characterized by extensive central necrosis, invasive growth and a more heterogeneous appearance. These characteristics were not visualized in this case, so was also not a sensible diagnosis. In addition to this differential diagnosis, peritoneal carcinomatosis were also less probable because of the absence of ascites and weight loss (Fasih et al., 2008).

In general, the treatment is a resection: open, laparoscopic or robotic, which depends on the location, the size of the mass and its connection to other organs (Grover and Ballha, 2015, Salih et al., 2017). Another possible option is the use of gonadotropin-releasing hormone agonists, which inhibits gonadal function and decreases serum oestrogen, and has been shown to produce a 40–60% reduction in the size of leiomyomata (Al-Wadaani, 2012). Other reported treatment modalities include: careful observation, myomectomy, hysterectomy with bilateral oophorectomy and medical treatments such as progestin and aromatase inhibitors (Mahmoud et al., 2015).

In the case reported, there was no need for hysterectomy nor ovariectomy since the tumour was completely isolated from the female genital organs.
